# Integrative analysis of TRPV family to prognosis and immune infiltration in renal clear cell carcinoma

**DOI:** 10.1080/19336950.2022.2058733

**Published:** 2022-04-07

**Authors:** Zixuan Gong, Jiaheng Xie, Liang Chen, Qikai Tang, Yiming Hu, Aiming Xu, Zengjun Wang

**Affiliations:** a Department of Urology, The First Affiliated Hospital of Nanjing Medical University, Nanjing, Jiangsu, China; b Department of Burn and Plastic Surgery, The First Affiliated Hospital of Nanjing Medical University, Nanjing, Jiangsu, China; c Department of General Surgery, Fuyang Hospital Affiliated to Anhui Medical University, Fuyang, Anhui, China; d Department of Neurosurgery, The First Affiliated Hospital of Nanjing Medical University, Nanjing, Jiangsu, China; e College of Pharmacy, Jiangsu Ocean University, Lianyungang, Jiangsu, China

**Keywords:** Clear cell renal cell carcinoma, transient receptor potential channels, bioinformatics, immune microenvironment, prognostic analysis

## Abstract

The transient receptor potential vanilloid (TRPV) family has been preliminarily discovered to play an important role in various cancers, including clear cell renal cell carcinoma (ccRCC), which is closely associated with immune infiltration. However, the expression and prognosis of TRPV family and tumor-infiltrating immune cells in ccRCC are obscure. This study aimed to explore the prognostic and therapeutic value of the TRPV family expression in ccRCC from the perspective of bioinformatics. We analyzed the transcriptome and clinical data of kidney renal clear cell carcinoma (KIRC) from The Cancer Genome Atlas (TCGA) database. A clustering analysis and immune infiltration analysis were conducted to investigate the influence of the TRPV family genes on ccRCC. Our study found that the TRPV family is an excellent prognostic stratification for ccRCC. Among them, TRPV3 is the most significant prognostic marker of ccRCC. In addition, we performed a drug sensitivity analysis to identify the drugs with the strongest association with TRPV3. As a result, the TRPV family, particularly TRPV3, can act as a prognostic biomarker in ccRCC to determine prognosis and levels of immune infiltration.

## Introduction

The 2021 Nobel Prize in Physiology or Medicine has been awarded to Professor David Julius for their work on transient receptor potential (TRP) pathways [1]. Thus, we can see the important significance of the TRP channels in human homeostasis and pathophysiology. Transient receptor potential channels participate in the regulation of intracellular physiological processes by recognizing various signals outside the cell, such as pressure, temperature, osmotic pressure, and substance composition [2–4]. In the course of cancer, the tumor microenvironment changes obviously [5]. These changes produce a cascade of intracellular amplification through various receptors. In this complex network, transient receptor potential channels may be important transporters [6,7]. The TRP channel family consists of seven subgroups: TRPV, TRPA, TRPC, TRPM, TRPML, TRPN, and TRPP [8]. Many studies have demonstrated that the TRP family is associated with excessive proliferation, activation of growth signals, and resistance to apoptosis in tumors [9,10]. Among them, the TRPV family has been studied more and has been found to be differentially expressed in many tumors [11].

The TRPV receptor family consists of six members, namely, TRPV1-6 [12]. TRPV1-4 has a low calcium ion dependence, whereas TRPV5 and TRPV6 have a high calcium ion dependence [13]. TRPV1 mainly exists in the plasma membrane of sensory neurons and is involved in the recognition of heat signals, pH changes, and chemicals (such as capsaicin) in the extracellular environment, playing an important role in the formation of pain and temperature sensations [14,15]. TRPV2 mainly exists in the inner membrane of cells and has been shown to mediate a variety of physiological processes, such as the perception of harmful stimuli, regulation of immune cell activity, and regulation of intracellular calcium homeostasis [16]. TRPV3 is widely expressed and is involved in the formation of the skin barrier, wound healing, temperature perception, pruritus, pain, and other processes [17]. Similarly, TRPV4 is widely expressed in a variety of tissues and can recognize thermal signals, changes in osmotic pressure, and mechanical stretching in the extracellular environment [18]. Significant osmotic dysregulation was shown in TRPV4-deficient mice [19]. TRPV5 and TRPV6 are two highly similar channel proteins with similar structures and functions [20]. Both these two channels are highly dependent on calcium ions and play a role in calcium homeostasis and calcium-dependent regulation of cellular functions [20]. TRPV5 is mainly expressed in the kidney, while TRPV6 is widely expressed in the kidney, prostate, placenta, and breast [21,22]. Although there have been more and more studies on TRPV receptors in recent years, our understanding of the TRPV receptor family in tumors is still far from enough, including their prognostic value and functional changes in tumors. Therefore, exploring the role of the TRPV family in tumors can provide new biological markers that are valuable in guiding patient prognosis and developing new treatments.

Renal clear cell carcinoma is the most common subtype of renal cancer and the most aggressive subtype [23]. Hypoxia, angiogenesis, metabolic reprogramming, and immune reediting are common in the tumor microenvironment of renal clear cell carcinoma [24]. At present, VEGF targeted therapy targeting angiogenesis and immune checkpoint inhibitors targeting tumor mutations have achieved good initial results in renal clear cell carcinoma [25]. However, a considerable number of patients with renal clear cell carcinoma still have low responses or drug resistance to treatment. Therefore, the discovery of new biomarkers for renal clear cell carcinoma is of great significance.

Bioinformatics is currently closely related to cancer research. Through bioinformatics, we can explore changes in the transcriptome and immune microenvironment of cancer and identify new biomarkers, thus providing references for precise treatment and prognosis assessment of cancer. Among them, the TCGA database is the most widely used, containing transcriptome data and clinical information for a variety of cancers.

In our study, we explored the clinical significance of the TRPV channel family in renal clear cell carcinoma. We performed expression analysis, survival analysis, regression analysis, immune cell infiltration analysis, etc. Our study can provide new ideas for the diagnosis and treatment of renal clear cell carcinoma.

## Methods

### Data download and processing

The UCSC Xena (http://xena.ucsc.edu/) website is a website that collects and organizes multiple tumor databases, including the well-known TCGA database. From this website, we downloaded kidney renal clear cell carcinoma (KIRC) transcriptome data of 607 samples, clinical characteristics data of 985 patients, and survival data of 979 samples from this website. A total of 526 tumor samples and 72 normal samples were obtained by eliminating duplicate samples and reserving primary tumor and para-cancer samples. Transcriptome data and clinical survival data of 522 renal clear cell carcinoma samples were obtained by matching patients’ transcriptome data with clinical survival data. For further analysis, the transcriptome expression data were log2 transformed.

### Clustering analysis

NMF (Non-negative matrix factorization) clustering analysis is a common way to divide and cluster samples. In this study, the R package “NMF” is used to cluster the samples. The number range of clustering is set as 2–10, the method is set as “Brunet,” the runs are set as 30, the random seed is set as 123,456, and then the samples are clustered. The optimal clustering number is determined by the maximum absolute value of the slope of the cophenetic graph.

### Immune infiltration analysis

Through a literature review, 28 kinds of marker genes for immune cells were collected. Single Cell Gene Set Enrichment Analysis (ssGSEA) analysis is a method to quantitatively score each sample based on the characteristic genes of a gene set. In this study, the scores of 28 immune cells in patients with renal clear cell carcinoma were quantified in this way.

### Mutation correlation analysis

The R package “MAfTools” is a powerful tool for analyzing genetic mutations. In this study, the “oncoplot” function was used to display the landscape of the top 20 mutated genes by integrating mutation data with clinical data, and the “somatic interactions” function was used to display the interrelationships between mutations of the higher-ranked genes. Finally, we calculated the tumor mutation load (TMB) of each sample using the TMB function of this R package.

### Weighted gene co-expression network analysis (WGCNA)

Weighted gene co-expression network analysis (WGCNA) is an algorithm that aggregates genes with co-expressed relationships by calculating the weights between genes. In this study, WGCNA was used to find module genes with relationships among different subtypes, and the optimal soft domain value was calculated by the pickSoftThreshold function. The minimum number of module genes was set to 100, and the deep split was set to 2 to obtain different modules. Finally, the labeled heatmap function was used to carry out a correlation analysis between modules and phenotype files.

### Gene enrichment analysis

Gene enrichment analysis was used to explore the enrichment of a group of genes in a gene function or pathway. The domain value was 0.05, and the enriched gene set entries with P < 0.05 were retained.

### Differential expression analysis and clinical correlation analysis

First, we explored the expression of TRPV family genes in KIRC and adjacent normal tissues and their relationship with clinical characteristics including gender, age, and tumor stage. Survival analysis was then used to explore their prognostic value. Diagnostic ROC analysis was then performed using the “evalmod” function of the “Precrec” package. Finally, the ROC curve for prognosis was calculated and drawn by the timeROC package. First, we explored the expression of TRPV family genes in KIRC and adjacent normal tissues and their relationship with clinical characteristics including gender, age, and tumor stage. Survival analysis was then used to explore their prognostic value. Diagnostic ROC analysis was then performed using the “evalmod” function of the “Precrec” package. Finally, the ROC curve for prognosis was calculated and drawn by the timeROC package.

### Drug sensitivity analysis

The CellMiner database (https://discover.nci.nih.gov/cellminer/) is based on a list of 60 cancer cells published by the National Cancer Institute’s Cancer Research Center (NCI). In this study, a group of chemical drugs related to the gene was obtained by matching the known genes in the database, and the drugs with P < 0.05 were retained.

### Statistical analysis

The rank-sum test was used for two or more groups of differences. The difference is statistically significant when a p-value is less than 0.05. The software used is R, version 4.0.5.

## Results

### NMF cluster analysis was performed in patients with KIRC

**Figure 1.**
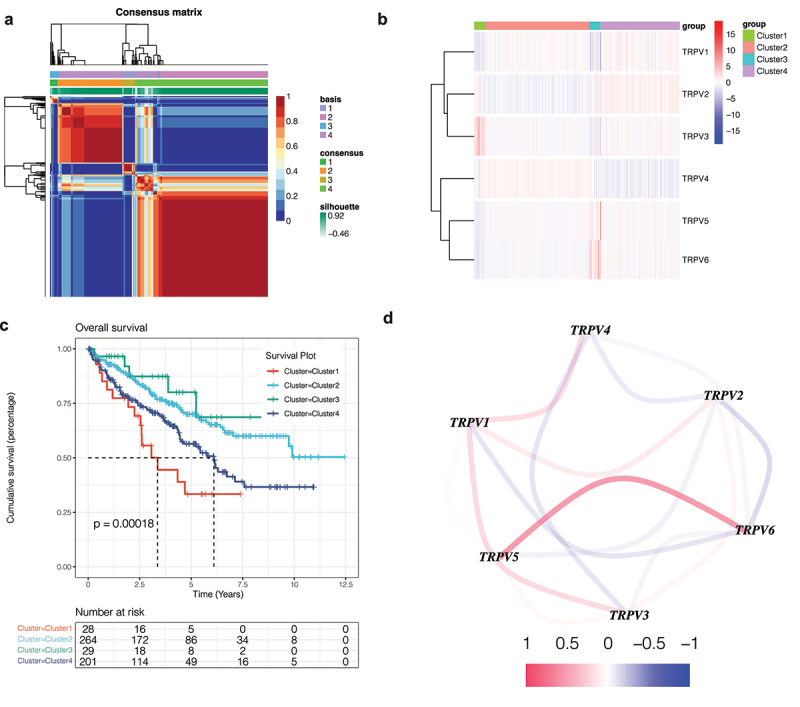
Non-negative matrix factorization (NMF) cluster analysis. (a) Kidney renal clear cell carcinoma (KIRC) patients can be divided into four clusters, respectively cluster 1, cluster 2, cluster 3, and cluster 4. (b) Heatmap of the expression of the TRPV family in different clusters. The darker the red, the higher the gene expression, and the darker the blue, the lower the gene expression. (c) Survival curve of different clusters. The prognosis of these clusters is different (P < 0.001). The prognosis for Cluster 1 is poor, while that for Cluster 3 is better. (d) The interaction network of TRPV family members. The relationship between TRPV5 and TRPV6 was the strongest and positively correlated, while that between TRPV3 and TRPV1 was positively correlated and that between TRPV3 and TRPV4 was negatively correlated.

In order to identify the influence of this family on KIRC, NMF cluster analysis was conducted on KIRC patients based on the expression of TRPV family genes. The results show that when the optimal number of clusters is 4, the absolute value of the slope of the cophenetic graph is the maximum. As shown in Figure 1(a), we found that KIRC patients were obviously divided into four clusters, namely, clusters 1 and 2, cluster 3, and cluster 4. Then, in order to further explore the gene expression of this family in different clusters, a heatmap was drawn (Figure 1(b)). The darker the red, the higher the gene expression, and the darker the blue, the lower the gene expression. In order to compare whether the prognosis of the four clusters is different, we drew the survival curve of the overall survival and found that the prognosis of these clusters is different (Figure 1(c), p<0.001). Moreover, we found that the prognosis of Cluster 1 is poor, while that of Cluster 3 is better. Subsequently, in order to observe the interaction between the TRPV family, we drew the interaction network diagram (Figure 1(d)) and found that the relationship between TRPV5 and TRPV6 was the strongest and positively correlated, while that between TRPV3 and TRPV1 was positively correlated and that between TRPV3 and TRPV4 was negatively correlated.

### Immune infiltration analysis

**Figure 2.**
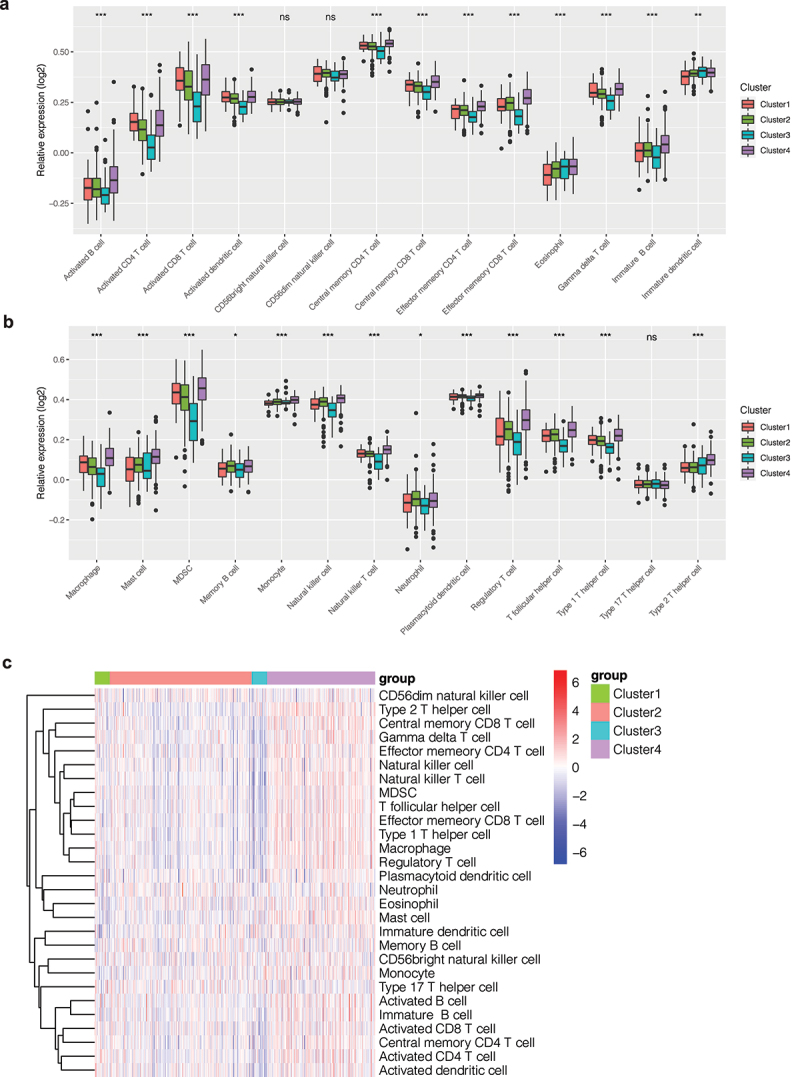
Immune infiltration analysis. (a, b) Bar chart of the immune infiltration level of different clusters. The infiltration level of most immune cells was different among different clusters (P < 0.05), such as activated B cells, activated CD4 T cells, activated CD8 T cells, etc. (c) Heatmap of immune infiltration in different clusters.

Different levels of immune infiltration may affect patient outcomes. To explore the differences in immune infiltration levels among different clusters, we quantified the scores of 28 types of immune cells in KIRC patients by the ssGSEA method. As shown in Figure 2(c), we found that scores of immune cells were different among different clusters. In Cluster 4, scores of immune cells were higher, while in Cluster 3, scores of immune cells were lower. In order to observe such differences, we drew a bar chart (Figure 2(a,b) and found that the infiltration level of most immune cells was different among different clusters (P < 0.05), such as activated B cells, activated CD4 T cells, activated CD8 T cells, etc. In addition, these immune cells showed a tendency for low expression in Cluster 3 and high expression in Cluster 4. In cluster 1, the infiltration level of many immune cells was higher than that of cluster 3. This difference in immune cell infiltration may account for the difference in prognosis between clusters.

### Gene mutation landscape

**Figure 3.**
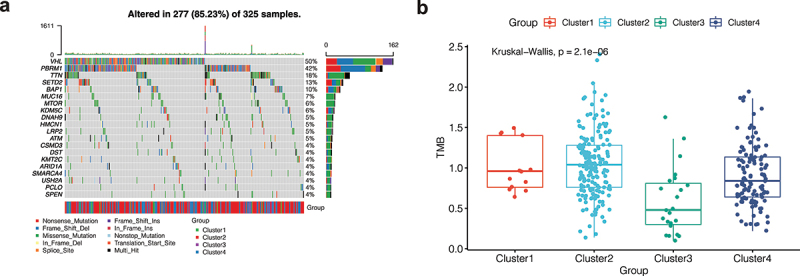
Mutation analysis. (a) The mutation landscape of KIRC. The highest mutation frequencies were VHL and PBRM1. Nonsense Mutation, Frame Shift Del, and Missense Mutation were the most common mutation types in the VHL gene. (b) The mutation symbiosis of the first 20 mutated genes. There was mutation symbiosis between PBRM1 and VHL (P < 0.01). Mutation symbiosis also existed between PBRM1 and SETD2, PBRM1 and USH2A, HMCN1 and TTN, LRP2 and SETD2, MUC16 and BAP1, USH2A and KDM5C, ARID1A and DNAH9, as well as USH2A and CSMD3 (P < 0.05). (c) Differential analysis of tumor mutation load (TMB) among different clusters. There was a significant difference in TMB among different clusters (P < 0.001), and the tumor mutation load in Cluster 3 was low.

As shown in Figure 3(a), the mutation landscape can intuitively see gene mutation in KIRC. There were 277 (85.23%) KIRC patients with mutations, among which the first two genes with the highest mutation frequency were VHL and PBRM1. Nonsense Mutation, Frame Shift Del, and Missense Mutation were the most common mutation types in the VHL gene. We further analyzed the mutation symbiosis of the first 20 mutated genes and found that there was a mutation symbiosis between PBRM1 and VHL (P < 0.01, Figure 3(b)). PBRM1 and SETD2, PBRM1 and USH2A, HMCN1 and TTN, LRP2, and SETD2, MUC16 and BAP1, USH2A and KDM5C, ARID1A and DNAH9, and USH2A and CSMD3 all had mutation symbiosis (P < 0.05, Figure 3(b)). At the same time, we conducted the differential analysis of tumor mutation load (TMB) among different clusters and found that there was a significant difference in TMB among different clusters (P 0.001, Figure 3(c)), and the tumor mutation load in Cluster 3 was low, suggesting that fewer mutations may occur in Cluster 3. This may be a factor in the better prognosis of cluster 3 patients.

### Weighted co-expression network analysis

**Figure 4.**
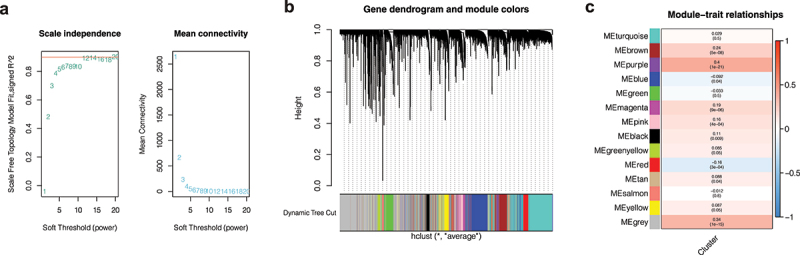
Weighted co-expression network analysis. (a) A soft domain value of 5 is optimal. (b) A total of 14 modules were obtained, and the proportion of gray modules was small. (c) The purple module was most correlated with the cluster.

As shown in Figure 4(a), when the soft domain value is set to 5, R2 on the left is greater than 0.8, indicating that the data is in line with the power-law distribution. Moreover, when the soft domain value is 5, the downward trend tends to be gentle. By setting the minimum module gene to 100 and the deep split to 2, we get a total of 14 modules (Figure 4(b)), and the proportion of gray modules is small. In order to find co-expressed module genes associated with different clusters, we conducted a joint analysis of module and cluster phenotype files and found that the purple module was most correlated with the purple cluster (COR = 0.4, P < 0.001, Figure 4(c)). We then selected a total of 267 genes from the purple module for subsequent analysis.

### Gene enrichment analysis

**Figure 5.**
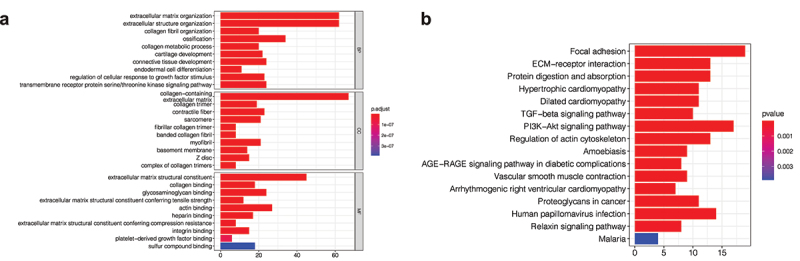
Gene enrichment analysis. (a) Gene ontology (GO) enrichment analysis. The enrichment results of purple module genes were mainly divided into the biological process (BP), cellular component (CC), and molecular function (MF). (b) KEGG enrichment analysis of purple module genes revealed that they were enriched in multiple pathways.

In order to further explore the functional characteristics and pathways of purple module genes related to the cluster, Gene enrichment analysis was performed on 267 genes in the purple module, and it was found that these genes were mainly related to extracellular matrix structural components and protein binding (Figure 5(a)). Furthermore, pathway analysis revealed that these genes were primarily associated with cell adhesion pathways and cancer-related pathways, such as the TGF- and PI3k-Akt pathways (Figure 5(b)).

### Correlation analysis of clinical features

**Figure 6.**
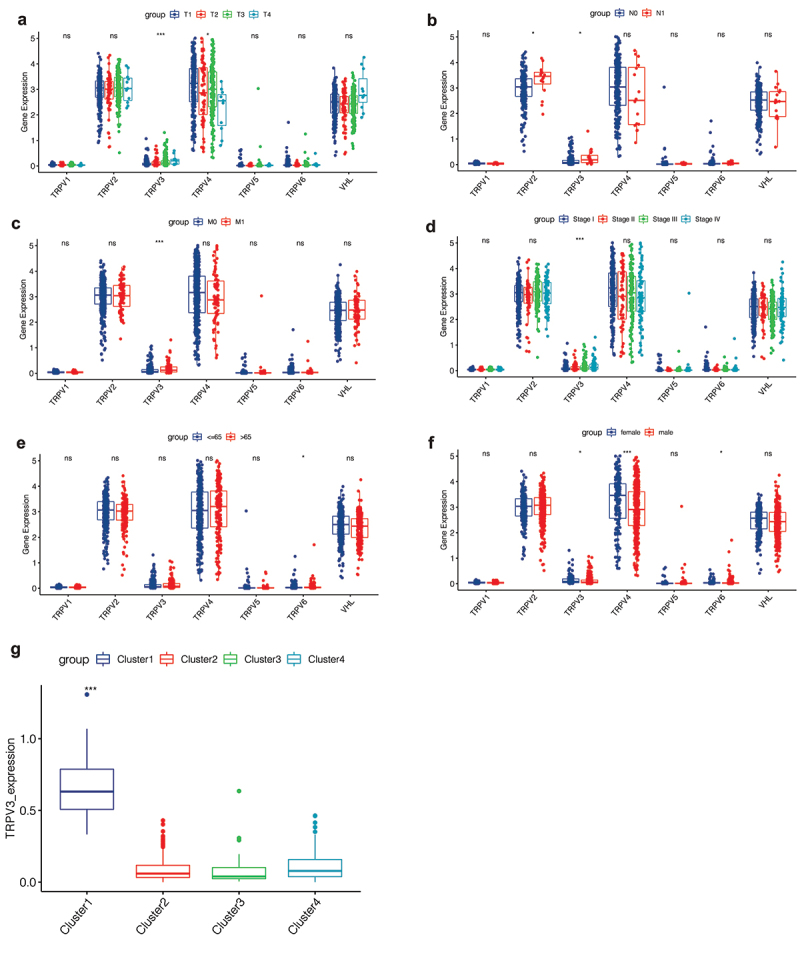
Correlation analysis of clinical features. (a) The expression levels of TRPV3 and TRPV4 are significantly different in patients with different T stages. (b) The expression levels of TRPV2 and TRPV3 are significantly different in patients with different N stages. (c) The correlation between the TRPV family and the M stage, and the results show that only TRPV3 is significantly correlated with different M stages. (d) The correlation between the TRPV family and total stage, and the results showed that only TRPV3 was significantly correlated with different total stages. (e) The relationship between the TRPV family and age (with 65 years as the cutoff value). Only TRPV6 was significantly associated with the two age groups. (f) The relationship between the TRPV family and gender, and the results showed that TRPV3, TRPV4, and TRPV6 were correlated with different genders. (g) TRPV3 was significantly overexpressed in cluster 1. Therefore, TRPV3 can help define cluster 1.

By matching the expression profiles of TRPV family genes with the clinical data, we explored the correlation between TRPV family genes and clinical features. Besides, we selected the well-known VHL gene, obviously related to ccRCC, as the control. Figure 6(a) shows the differences in the T stage, in which the expression levels of TRPV3 and TRPV4 are significantly different in patients with different T stages. In Figure 6(b), we explored the relationship between the TRPV family and the N stage. Figure 6(c) shows the correlation between the TRPV family and the M stage, and the results show that only TRPV3 is significantly correlated with different M stages. Subsequently, Figure 6(d) showed the correlation between the TRPV family and total stage, and the results showed that only TRPV3 was significantly correlated with different total stages. Then, we explore the relationship between the TRPV family and age (with 65 years as the cutoff value). Only TRPV6 was significantly associated with the two age groups (Figure 6(e)). Finally, we analyzed the relationship between the TRPV family and gender, and the results showed that TRPV3, TRPV4, and TRPV6 were correlated with different genders (Figure 6(f)). However, the VHL gene was not differentially expressed in different stages, ages, and gender groups. Of the six members of the TRPV family, TRPV3 is the most closely associated with clinical features. TRPV3 was differentially expressed in different T stages, N stages, M stages, total stages, and genders of ccRCC. Therefore, TRPV3 can be regarded as a robust clinical biomarker for ccRCC. The we conducted correlation analysis between TRPV3 and clusters (Figure 6(g)), and the results showed that TRPV3 was significantly overexpressed in cluster 1. Therefore, TRPV3 can help define cluster 1.

### Survival analysis and construction of diagnostic ROC curve

**Figure 7.**
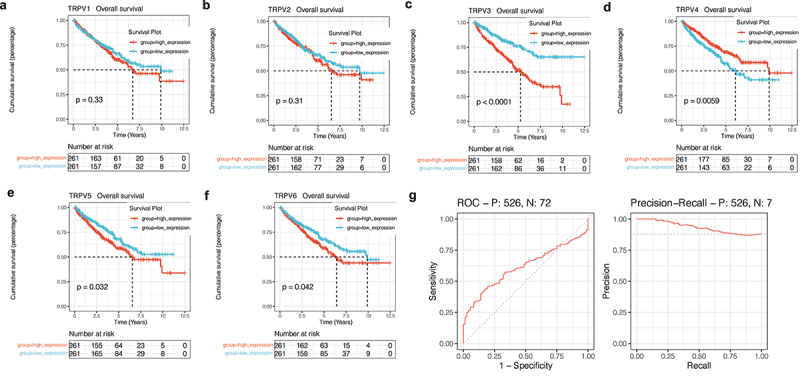
Survival analysis and construction of diagnostic ROC curve. (a-f) The survival analysis of the TRPV family in KIRC. The upregulation of TRPV3, TRPV5, and TRPV6 significantly reduced the overall survival rate of KIRC patients (P < 0.05). However, upregulation of TRPV4 expression was associated with a better prognosis (P < 0.05). (g) It was found that TRPV3 in TRPV family had a high diagnostic value for prognosis, and the AUC value fluctuated around 0.7.

Next, survival analysis was used to explore the prognostic value of the TRPV family in KIRC. As shown in Figure 7(a–f), the upregulation of TRPV3, TRPV5, and TRPV6 significantly reduced the overall survival rate of KIRC patients (P < 0.05). However, upregulation of TRPV4 expression was associated with a better prognosis (P < 0.05). Among them, the prognosis difference between the TRPV3 high expression group and the low expression group was the most significant (Figure 7(c)). Therefore, the effect of TRPV3 on prognosis may be stronger. At the same time, we also conducted ROC analysis of TRPV3 related to prognosis. It was found that TRPV3 in the TRPV family had a high diagnostic value for prognosis, and the AUC value fluctuated around 0.7 (Figure 7(g)).

### Drug sensitivity analysis

**Figure 8.**
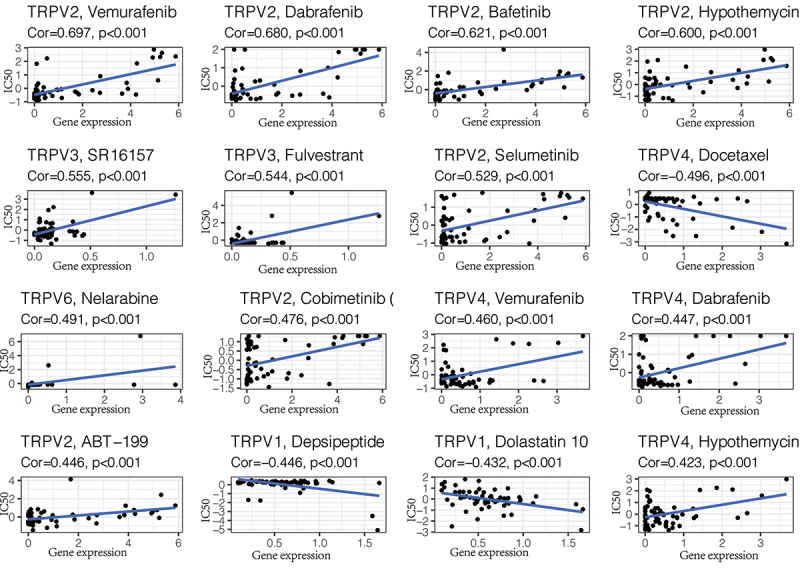
Drug sensitivity analysis. The top 16 predicted chemical drugs related to the TRPV3 in KIRC. Among them, SR16157 and Fulvestrant had the strongest correlation with TRPV3 (P < 0.001).

The above analysis showed that TRPV3 is the member with the strongest correlation with ccRCC in the TRPV family. Its high expression is associated with a poor prognosis of ccRCC, so targeting TRPV3 therapy may have potential therapeutic value. To further screen drugs related to TRPV3, we conducted drug sensitivity analysis through the cellMiner database, screened out drugs with P < 0.05, and arranged them in descending order of relevance. The top 16 predicted chemical drugs are shown in Figure 8. Among them, SR16157 and Fulvestrant had the strongest correlation with TRPV3 (P < 0.001). This provides a theoretical basis for subsequent drug research and is conducive to promoting the drug treatment of ccRCC.

## Discussion

With the rapid advancement of genomics in the 21st century, the treatment of renal clear cell carcinoma has entered a new era [26]. A novel path for targeted treatment of renal clear cell carcinoma has emerged due to VEGF mutations and aberrant PI3K/AKT/mTOR signaling [27]. Immune checkpoint inhibitors have become a prominent therapy option for individuals with renal clear cell carcinoma. Unfortunately, drug resistance is prevalent, resulting in a considerable number of deaths, despite the fact emerging therapies can enhance patient survival to some extent [28]. As a result, it is necessary to explore potential biomarkers and develop novel treatment regimens.

Calcium disorders are frequent in a variety of cancers. Calcium ions are one of the most effective second messengers in cells, so their disruption could affect the cells’ ability to proliferate and invade [29]. Calcium disturbance may also be the key mechanism of the malignant transformation of tumors [30, 31]. The importance of calcium channel research in cancer is self-evident. The TRPV receptor family is the most studied subset of the transient potential receptor family, and it is involved in calcium homeostasis regulation [12]. TRPV receptors have been implicated in pain, inflammation, and wound healing in previous research. However, few studies on TRPV receptors in renal clear cell carcinoma are currently available. It is necessary to illustrate the potential role of the TRPV family in renal clear cell carcinoma.

In this study, we identified the importance of TRPV family members in renal cancer by integrating transcriptome and clinical data. The research methods herein include cluster analysis, immune infiltration analysis, mutation analysis, weighted gene co-expression analysis, enrichment analysis, and drug sensitivity analysis. This not only adds to our understanding of the tumor microenvironment but also offers a novel set of biomarkers and drug targets for renal clear cell carcinoma.

At present, previous studies have suggested that the TRPV family may have a role in cancer. Gao et al. found that TRPV1 inhibits GC development via a novel Ca2+/CaMKK/AMPK pathway [32]. TRPV2 is implicated in the migration of prostate cancer cells, according to Monet et al., indicating that TRPV2 might serve as a potential prognostic marker and therapeutic target for advanced prostate cancer [33]. Li et al. found that TRPV3 is overexpressed in NSCLC, with association with lung cancer growth and development [34]. TRPV4 promotes endometrial cancer cell migration and is linked to a poor outcome in endometrial cancer, according to Li et al [35]. Reduced TRPV5/6 expression was associated with a poor prognosis in NSCLC, according to Fan et al [36]. In addition, the TRPV family’s significance in renal cell cancer has been investigated. According to Wu et al., TRPV1 expression was observed to have a substantial correlation with tumor Fuhrman grade and histological subtypes in RCC [37]. By mediating the expression of the TRPV5/6 channel, Chen et al. discovered that altered vitamin D receptor expression may be associated with renal cell carcinoma [38]. The underlying mechanisms and functions of the TRPV family in cancer remain poorly understood and require further study.

Our study identified TRPV3 as a robust prognostic marker of ccRCC by comprehensive analysis of the TRPV family. The prognosis of ccRCC patients with high TRPV3 expression was significantly worse. In addition, many clinical characteristics of patients with TRPV3 and ccRCC were correlated, such as T stage, N stage, M stage, total stage, and gender. TRPV3 was also found to be significantly upregulated in cluster 1 with the worst prognosis. This revealed that TRPV3 is a new biomarker of ccRCC and can be beneficial for the diagnosis and treatment of ccRCC.

In conclusion, the TRPV family may be investigated further in clear cell renal carcinoma. They might have a key role in tumor initiation, promotion, and development in ccRCC. Our study can favor the diagnosis and treatment of clear-cell renal carcinoma, as well as the exploration of the immune microenvironment. Nevertheless, several deficiencies exist in this study. We lack relevant experiments to verify our conclusions, and we will make improvements in the future.

## Conclusion

Our study is the first to confirm the significance of the TRPV family in renal clear cell cancel from the perspective of bioinformatics. The findings can provide a useful reference for the diagnosis and treatment of renal carcinoma as well as the investigation of the immunological microenvironment.

## Supplementary Material

Supplemental Material

## Data Availability

The data that support the findings of this study are openly available in the ICGC database at [http://xena.ucsc.edu/].
